# Retrospective review of in hospital use of mineralocorticoid receptor antagonists for high risk patients following myocardial infarction

**DOI:** 10.1186/s12872-015-0033-1

**Published:** 2015-06-10

**Authors:** Robert J. H. Miller, Jonathan G. Howlett

**Affiliations:** Libin Cardiovascular Institute of Alberta, University of Calgary, Room C-838, 1403-29th Street NW, Calgary, T2N 2Y8 AB Canada

**Keywords:** Heart failure, Myocardial infarction, Mineralocorticoid receptor antagonists

## Abstract

**Background:**

There is little data regarding use of mineralocorticoid antagonists (MRAs) for patients reduced LV ejection fraction (LVEF) following acute myocardial infarction (MI). We determined the frequency and temporal trends of MRA use in these patients.

**Methods:**

We performed a retrospective review of all cases of acute MI between June 1, 2010 and April 1, 2012. Patients were considered eligible for MRA therapy if they were admitted with acute MI with LVEF ≤ 40 % and had heart failure symptoms or a history of diabetes.

**Results:**

Of 3910 cases of acute MI, 332 patients were considered eligible for MRA therapy. MRA therapy was prescribed for 92/332 (28 %) eligible patients, while 66 of 1142 (6 %) of ineligible patients were so treated. Over the study period, usage in eligible and ineligible patients rose significantly (22 to 30 %, *p* = 0.08 and 4 to 7 %, *p* = 0.04 respectively).

**Conclusions:**

Prescription of MRAs for eligible patients occurred in a minority of patients, and demonstrated a modest increase over time. In patients without an indication for MRAs, a similar trend was observed. Further study is required to better understand barriers to appropriate use of MRAs in this patient population.

## Background

Pharmacologic interventions such as angiotensin converting enzyme (ACE) inhibitors, angiotensin receptor antagonists (ARBs) and beta blockers are known to benefit patients with heart failure (HF) and reduced left ventricular ejection fraction (LVEF) complicating acute myocardial infarction (MI). In 2003, following publication of the Eplerenone Post-Acute Myocardial Infarction Heart Failure Efficacy and Survival Study (EPHESUS), mineralocorticoid receptor antagonist (MRA) therapy was included in this list [1].

The use of MRA therapy in patients with HF and reduced LVEF is not new. Following the publication of the Randomized Aldactone Evaluation Study (RALES) trial, use of spironolactone was considered standard therapy for patients with moderate to severe HF and LVEF < 35 % [2]. However, registries have shown a low uptake in MRA therapy for patients with post-MI and chronic HF [3,4]. Following the RALES trial, spironolactone use increased from 3 to only 24 % of patients meeting enrollment criteria [3]. Interestingly, MRA use increased from 3 to 18 % in patients not meeting RALES enrollment criteria, with a concurrent increase in hospitalizations for hyperkalemia [3,5].

Evidence for MRA therapy in patients with reduced LVEF has been accumulating over time. Recent studies such as Eplerenone in Patients with Systolic Heart Failure and mild symptoms (EMPHASIS-HF) study showed a significant reduction in mortality with the addition of eplerenone to standard HF therapy in patients with mild to moderate symptoms and reduced LVEF [6]. Consequently, the Canadian Cardiovascular Society (CCS) recommended an MRA be considered for patients already on standard HF therapy who meet these enrolment criteria [7].

MRAs are thought to be underutilized, and at our centre we anecdotally noted a low rate of MRA prescriptions for post-MI patients with reduced LVEF. In the Canadian Heart Failure network, outpatient MRA therapy utilization approaches 38 % [8]. According to CIHI data, Calgary area hospitals report a mortality rate below the national median for MI [9]. This represented an opportunity to describe the rate of MRA prescription in a large cohort of post-MI patients who were presumably well treated following supportive publications for MRA use. In addition, temporal changes in prescription of MRAs could be assessed. We hypothesized that appropriate utilization of MRAs would be low with an increase in use over time.

## Methods

We completed a retrospective review of patients discharged alive with a diagnosis of acute MI (ICD-10 code I-21) from three Calgary metropolitan area hospitals between June 1, 2010 and April 1, 2012. We excluded patients who underwent hospital transfer and patients who did not have an assessment of LV function.

From this group of patients we collected the following clinical information: LVEF, type of MI (ST elevation (STEMI) or non-ST elevation MI (NSTEMI)), length of hospitalization, admission blood pressure, admission heart rate, and admitting service. We obtained past medical history of: HF, previous MI, hypertension, diabetes, dyslipidemia, and smoking. In addition the following laboratory information was collected: discharge serum creatinine, peak potassium and peak troponin. Glomerular filtration rate (GFR) was estimated using the MDRD equation [10]. Use of MRA (either spironolactone or eplerenone), beta-blockers, ACE-inhibitors and ARBs were assessed based on discharge summaries and electronic prescription records. LVEF was recorded in order of preference based on the 2D biplanar Simpson’s model, subjective estimated LVEF and subjective global impression.

Patients were considered eligible for MRA therapy if there was documented LVEF ≤ 40 % and symptoms of HF or a history of diabetes. Patients were considered ineligible if there was documented: allergy or intolerance to MRA therapy, a serum potassium level > 5.0 meq/L, estimated GFR <30 mL/min/1.73 m^2^, or documented patient refusal. These criteria were based on current ACC guidelines [11]. A standardized data extraction tool was used to minimize bias during the data collection process.

We explored temporal change by comparing prescribing rates of MRAs before (period A) and after (period B) the date of publication of EMPHASIS-HF trial (January 6, 2011) for both eligible and ineligible patients. This date was chosen to investigate the effects of a landmark trial publication on medication use. All data were reported using descriptive statistics with means and standard deviations for continuous variables and simple percentages for categorical variables. Fisher exact tests were used to analyze differences in proportions according to categorical stratifications. Linear regression was used to determine a trend in overall use with the least squares method after dividing usage rates by quarters, centered around the EMPHASIS-HF publication date. We performed a logistic regression analysis using the least squares method to determine factors associated with MRA prescription, with significance denoted by *p* < 0.05. Analyses were conducted using Stata version 13 (Stata Corp., College Station, Texas). Ethics approval was obtained from the University of Calgary Conjoint Health Research Ethics Board.

## Results

**Fig. 1:**
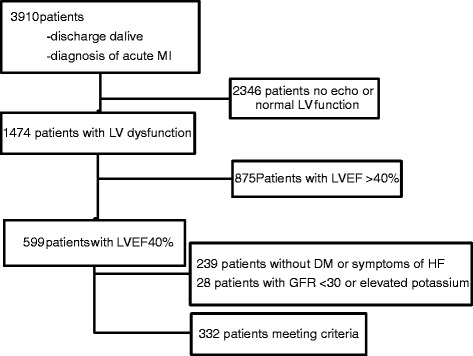
Study flow sheet outlining patient inclusion and exclusion. *DM* diabetes mellitus, *GFR* glomerular filtration rate, *HF* heart failure, *LV* left ventricle, *LVEF* left ventricular ejection fraction

**Fig. 2:**
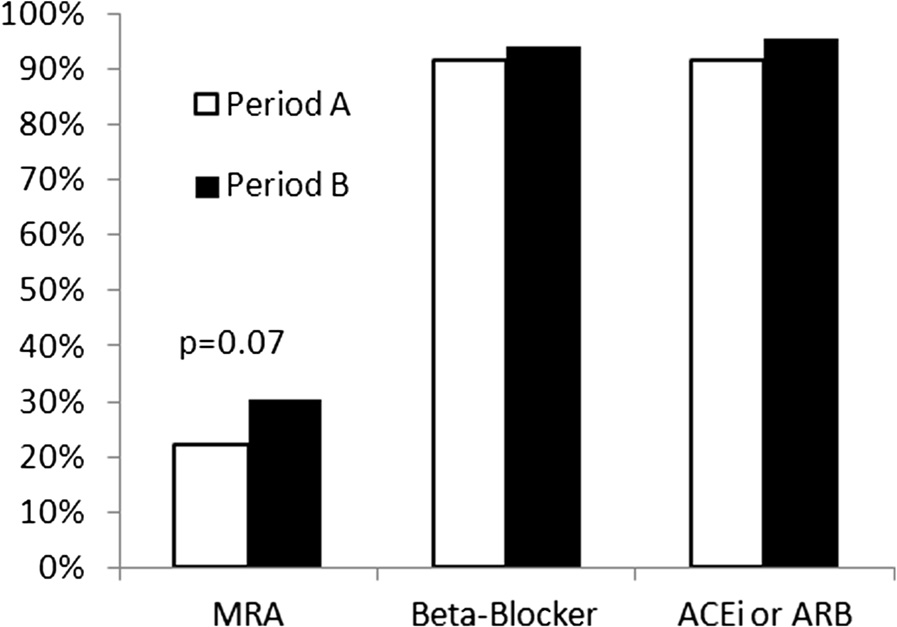
Prescriptions of MRA, beta-blockers, and ACE-inhibitors or ARBs in patients meeting criteria for MRA usage between study periods. *MRA* mineralocorticoid receptor antagonist, *ACE-i* angiotensin converting enzyme inhibitor, *ARB* angiotensin receptor blocker

We identified 3910 patients discharged alive with a diagnosis of acute MI, of whom 1474 had documented reduced LVEF. Five hundred ninety-nine patients had documented LVEF ≤ 40 %, of which 332 met our eligibility criteria for MRA therapy without exclusions. Patient characteristics are outlined in Table 1. Study flow sheet outlining patient inclusion is shown in Fig. 1. Overall, 92 of 332 eligible patients (28 %) were prescribed MRA therapy. This percentage was numerically lower during period A (24/108, 22 %) versus those discharged in period B (68/224 or 30 %, *p* = 0.07, see Fig. 2), however this was not statistically significant. If all prescriptions filled within 6 months of discharge were included, MRAs were prescribed in 33/108 (31 %) patients for period A and 79/224 (35 %) for period B (*p* = 0.23 between periods).

**Table 1:** Population characteristics for patients with documented systolic dysfunction

		Eligible (*n* = 332)	Ineligible (*n* = 1142)
Demographics	Male (%)	250 (75.3 %)	842 (73.7 %)
	Age (years)	67.6 +/− 12.8	64.7+/− 13.2
	Length of stay (days)	15 +/− 21	9 +/− 13
Medical history	Hypertension	199 (59.9 %)	624 (54.6 %)
	Dyslipidemia	132 (39.8 %)	407 (35.6 %)
	Diabetes	187 (56.3 %)	272 (23.8 %)
	Smoking	107 (32.2 %)	399 (34.9 %)
	Myocardial infarction	117 (35.2 %)	257 (22.5 %)
	Heart failure	86 (25.9 %)	77 (6.7 %)
Clinical data	Systolic blood pressure (mmHg)	114 +/−18	119 +/− 23
	Heart rate (beats/min)	78 +/− 15	72 +/− 14
	Ejection fraction	32.7 +/− 7.1	45.6+/− 6.9
	STEMI	141 (42.5 %)	637 (55.8 %)
Laboratory data	Peak troponin T (μg/L)	4.0 +/− 5.9	3.7+/− 4.9
	Peak potassium (mmol/L)	4.5 +/− 0.4	4.5 +/− 0.5
	Estimated GFR (mL/min/1.73 m^2^)	81 +/− 36	77 +/− 30

*EF* ejection fraction, *μg/L* micrograms per liter, *μmol/L* micromole per liter, *mmHg* millimeters mercury, *mmol/L* millimoles per liter, *STEMI* ST elevation myocardial infarction. All numerical values shown +/− standard deviation

**Fig. 3:**
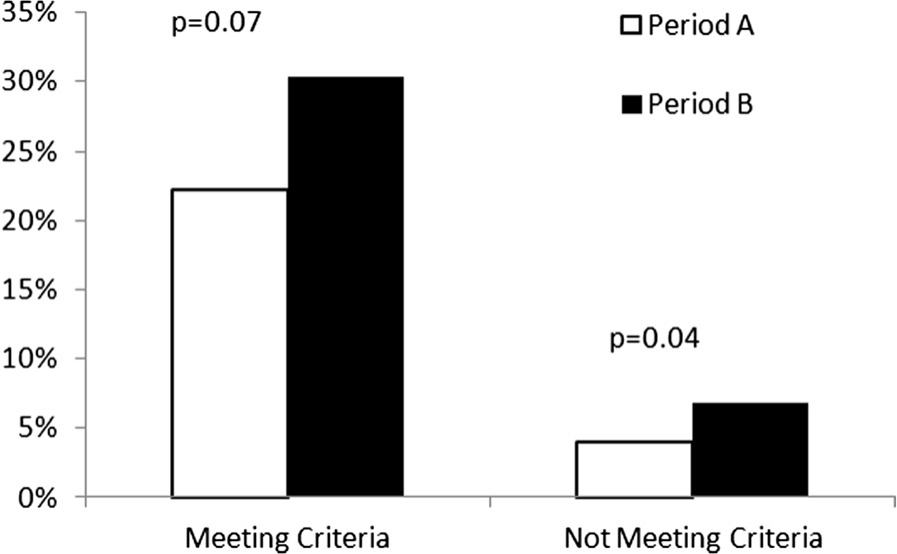
Use of MRAs in patients meeting and not meeting our criteria between study periods

We identified 1142 patients with systolic dysfunction who did not meet our criteria. In these patients, MRAs were prescribed in 16/401 (4 %) patients during period A and 50/741 (7 %) during period B (*p* = 0.04 between periods, see Fig. 3).

When considering only patients admitted to a cardiology service, 32 % were prescribed MRAs, with 16/71 patients (23 %) given during period A and 54/148 (36 %) for period B (*p* = 0.03). For patients not meeting our criteria the corresponding proportions were 14/323 (4 %) and 40/585 (7 %, *p* = 0.08). Prescribing rates between periods were not analyzed for other admitting services due to low patient numbers. Cumulative prescribing rates for eligible patients were; cardiovascular surgery 7/43 (16 %), family practice 7/33 (21 %), and internal medicine 6/18 (33 %). For ineligible patients, the rates of MRA prescription were: cardiovascular surgery 4/96 (4 %) family practice 4/58 (7 %) and internal medicine 3/36 (8 %). There were no significant differences in prescribing rates between admitting services.

**Fig. 4:**
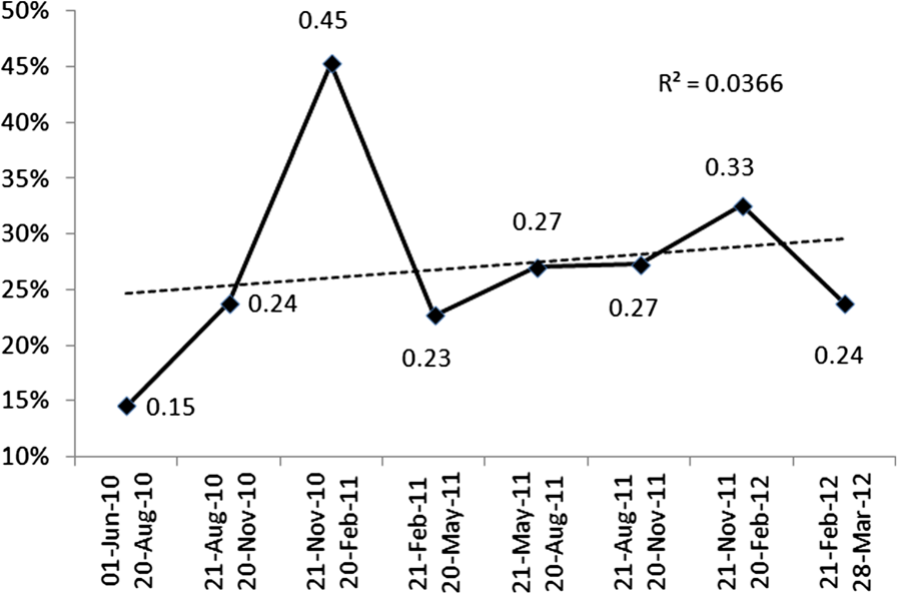
Proportion of patients using MRAs by quarter with overall trend in use

The proportion of eligible patients prescribed MRAs by quarter are displayed in Fig. 4. However the coefficient of determination (R^2^) was only 0.036 (*p* = 0.02). For comparison purposes, we also collected the prescription rates for other therapies with longstanding indications for patients with acute MI (see Fig. 1). Beta-blockers were prescribed at similar rates across periods (99/108, 92 % vs. 211/224, 94 %). There were similar findings for ACE-inhibitors and ARBs.

We performed a logistic regression analysis to identify factors associated with MRA prescriptions in both eligible and ineligible patients. We assessed the following possible associated factors: age, gender, length of hospitalization, history of HF, hypertension, diabetes, smoking, dyslipidemia, and previous MI, systolic blood pressure, heart rate, type of MI, EF, estimated GFR, peak troponin, and potassium. The results of this analysis are outlined in Table 2. In patients eligible for MRA therapy, lower EF, history of smoking, and history of dyslipidemia were associated with higher rates of MRA prescription (all *p* < 0.05). In patients who were considered ineligible for MRA therapy, lower EF and history of HF were associated with higher rates of MRA prescription (all *p* < 0.05).

**Table 2:** Logistic regression analysis to identify factors associated with MRA prescription

		Eligible		Ineligible	
		OR (95 % CI)	Adjusted *p*-value	OR (95 % CI)	Adjusted *p*-value
Demographics	Age	1.01 (0.98–1.03)	0.69	1.00 (0.98–1.02)	0.91
	Female	0.97 (0.51–1.83)	0.92	2.22 (1.27–3.88)	0.01
	Length of stay	1.01 (0.99–1.02)	0.33	1.01 (0.99–1.03)	0.17
Medical history	Heart failure	1.66 (0.83–3.32)	0.15	2.38 (0.97–5.85)	0.06
	Hypertension	0.99 (0.56–1.75)	0.97	1.24 (0.70–2.17)	0.46
	Dyslipidemia	0.47 (0.26–0.85)	0.01	0.73 (0.41–1.29)	0.40
	Diabetes	1.06 (0.61–1.83)	0.84	1.33 (0.69–2.56)	0.28
	Smoking	1.84 (1.03–3.27)	0.04	1.39 (0.81–2.39)	0.23
	MI	0.99 (0.50–1.95)	0.98	1.05 (0.54–2.03)	0.89
Clinical data	SBP	0.99 (0.97–1.00)	0.16	1.00 (0.99–1.01)	0.58
	Heart rate	1.01 (0.99–1.03)	0.17	0.99 (0.97–1.01)	0.40
	LVEF	0.93 (0.90–0.97)	0.00	0.93 (0.90–0.96)	0.00
	STEMI	1.44 (0.74–2.80)	0.28	1.62 (0.85–3.10)	0.15
Laboratory data	Troponin T	1.02 (0.97–1.07)	0.39	1.05 (1.00–1.09)	0.05
	Potassium	0.50 (0.23–1.08)	0.08	1.01 (0.56–1.79)	0.99
	Estimated GFR	1.00 (0.99–1.01)	0.87	1.00 (0.99–1.01)	0.74

Analysis of factors associated with increased rates of MRA prescription. *CI*, confidence interval; *GFR*, glomerular filtration rate; *LVEF*, left ventricular ejection fraction; *μg/L*, micrograms per liter; *μmol/L*, micromole per liter; *mmHg*, millimeters mercury; *mmol/L*, millimoles per liter; *OR*, odds ratio; *STEMI*, ST elevation myocardial infarction; *SBP*, systolic blood pressure

## Discussion

We had hypothesized that MRA prescription would be suboptimal in eligible patients with reduced LVEF following acute MI. Over time, there was a trend towards an increase in the utilization of MRA therapy for both eligible and ineligible patients, although this was not statistically significant in patients eligible for MRA therapy. Overall, prescribing rates were significantly lower than we found for beta-blockers and ACE-inhibitors or ARB’s. For these agents we found a very high usage rate which did not change over time, as one might expect of an established standard of care. We’ve shown that across three medical centers where overall survival for MI is better than the norm, there is a low rate of MRA usage [9]. Indeed, this level is below that seen in other jurisdictions, such as in Madrid, Spain (50 %), [12] and in many US hospitals [4].

Previous studies have identified suboptimal use of MRA therapy for patients with HF and reduced LVEF, but have not, until recently, reported usage rates of MRAs in post-MI patients with low LVEF [13,14]. While there has been a clear lack of emphasis on MRA usage in eligible patients following acute MI, reports regarding the usage in chronic HF have hypothesized a lack of confidence in diagnosis, concerns regarding medication use in fragile patients, poor awareness of research evidence and individual preference as barriers in HF management [15,16]. Additionally, some physicians may feel that adherence to guidelines does not change clinical outcomes, which may be true specifically for MRA therapy [15]. While there was a trend towards an increase in prescriptions between periods this seemed to reflect an overall upward trend in usage. This suggests a role for ongoing educational efforts such as education at the time of guideline implementation, continuing education, and audit-feedback systems [16].

While we did find an increase in appropriate prescriptions over time, we also found a smaller, but statistically significant increase in use in patients not meeting our criteria for MRA usage. We identified EMPHASIS-HF as a landmark trial in MRA therapy that may have renewed enthusiasm for this class of medications even though it investigated a different patient population then our current study [6]. We did see an increase in patients we deemed ineligible for MRA therapy which may reflect a direct impact from this publication or a more global trend towards increased use. Interestingly, there were similar findings following publication of the RALES trial, suggesting that landmark trials have important effects outside of their studied populations [3]. Therefore, it may be of benefit to specifically outline both inclusion and exclusion criteria in major studies, guidelines and educational efforts to optimize clinical decision making.

Our study had several important limitations. Due to the retrospective nature of our study, only data that was collected for clinical purposes was recorded. This was a single-center experience limiting the generalizability of the results and the small sample size limited our ability to detect a difference in prescribing rates, particularly between admitting services. Our exclusion criteria were more restrictive than would typically be used in clinical practice. This would have lead to a falsely high usage rate, making the low use found in our study more significant. Finally, in the EPHESUS study, patients were randomized to eplerenone therapy at an average of 7.3 days [1]. It’s possible that patients were discharged before MRA therapy could be initiated, however the low rates of prescriptions in follow-up and lack of impact of length of hospitalization on utilization argues that this is not the case.

## Conclusions

Despite its limitations, our study highlights some important considerations. Use of MRAs has increased, but they continue to be underutilized. Further efforts to improve the appropriate usage of MRAs are required. Finally, interventions other than publication of landmark trials are likely required to accelerate optimal usage of MRAs in eligible patients.
